# CONTRACT NURSE UTILIZATION AND FACILITY AND COMMUNITY FACTORS

**DOI:** 10.1093/geroni/igad104.0745

**Published:** 2023-12-21

**Authors:** Rohit Pradhan, Ganisher Davlyatov, Neeraj Dayama, Robert Weech-Maldonado

**Affiliations:** Texas State University, San Marcos, Texas, United States; University of Oklahoma Health Sciences Center, Oklahoma City, Oklahoma, United States; Texas Tech University Health Sciences Center, Lubbock, Texas, United States; University of Alabama at Birmingham, Birmingham, Alabama, United States

## Abstract

While NHs have long been plagued by nursing staff shortages, the situation has further deteriorated with the COVID 19 pandemic. The NH industry has attempted to address the persistent shortage of nursing staff by increasingly relying on contract nurse staffing. Research suggests that the increased utilization of contract nurses may have negative implications for NH quality and financial performance. However, all NHs are not equally likely to utilize nurse contract staffing. For instance, potentially, NHs with poorer quality of care and financial challenges may be forced to rely more on contract nurse staffing. The primary purpose of this paper is to explore the antecedents---facility and community factors---associated with increased utilization of nurse contract staffing. Employing a pooled cross-sectional observational study design, we extracted secondary data from the PBJ nurse staffing, Nursing Home Compare, Rural-urban commuting area (RUCA) codes, and the American Community Survey (ACS) for the period 2017-2021. To test the relationship between the ratios of contract nurses and facility/community factors, we employed multivariate mixed-effects maximum likelihood regression. We ran three separate regressions for RNs, LPNs, and CNAs. Our results suggest that among facility level factors, NHs with more beds, higher occupancy rates, and higher proportion of Medicare residents were generally more likely to employ contract nurse staffing with some minor differences across the three models (RNs, LPNS, CNAs). Among community factors, location in micropolitan/small town areas, higher poverty rates, and higher education levels were positively associated with nurse contract utilization. Policy and managerial implications are discussed.

